# The Relationship Between Hope and Religious Coping Among Patients With Type 2 Diabetes

**DOI:** 10.5539/gjhs.v8n1p208

**Published:** 2015-05-20

**Authors:** Abbas Shamsalinia, Tayebe Pourghaznein, Marzie Parsa

**Affiliations:** 1 School of Nursing and Midwifery, Babol University of Medical Sciences, Ramsar, Mazandaran, Iran; 2 PhD Student in Nursing, School of Nursing and Midwifery, Mashhad University of Medical Sciences, Mashhad, Iran; 3 Shahid Madani Hospital, Icu Ward, Karaj, Iran

**Keywords:** hope, religious coping, Type 2 diabetes

## Abstract

**Background and Purpose:**

Globally, diabetes is one of the most common non-contagious diseases resulting in severe complications. Fostered hope facilitates coping and improves self-care and one of the Factors affecting hope is religious beliefs. This research investigated the level of hope and its relationship with religious coping among Type 2 diabetes patients.

**Material and Methods:**

This correlation, cross-sectional study was conducted on 150 patients with Type 2 diabetes, who had been referred to the Karaj Diabetes Association during the period, March–June 2011, and selected through purposive sampling. A three-part questionnaire including demographic data, the Herth Hope Index, and a short form of religious coping, was used for data collection. The data were analyzed using descriptive and analytic statistics, including Pearson’s correlation coefficient, the *t*-test, a one-way ANOVA, and a multiple regression analysis. The set significance level was p<0.05.

**Results:**

The mean hope score was 34.89 (SD±8.75); most of the subjects (46.7%) showed high levels of hope. Positive religious coping, marital status, and social support significantly affected hope fostering (r=0.897, p =0.000). A significant negative relationship was found between hope and age (r=-0.373, p=0.000), and between hope and negative religious coping (r=-0.749, p=0.000).

**Conclusion:**

Positive religious coping, married life, and social support significantly affected the development of hope. Moreover, there was a significant positive relationship between positive religious coping and social support. So, strengthening social support could lead to increased levels of positive religious coping and fostering of hope.

## 1. Introduction

Diabetes is one of the most common, non-contagious diseases that lead to severe complications, with a growing prevalence across the world (Group, 2003; Strine, Okoro, Chapman, Beckles, Balluz, & Mokdad, 2005). The World Health Organization (WHO) has estimated that, in 2030, 360 million people will be suffered from diabetes (Kartal & Özsoy, 2007). The incidence of diabetes in Iran is currently estimated at more than 4 million. Diabetes results in several severe and, sometimes, irreversible complications (Funnell et al., 2009). Various studies have shown the importance of medical interventions and self-care practices in preventing complications (Association, 1993; Eastman & Keen, 1997; Group, 1998). Despite these interventions, the incidence of diabetes complications is remained high; this could be due to failure in following the therapeutic regimen and engaging in self-care behaviors (Tan, 2004), as most patients lack sufficient motivation to undertake self-care and exercise meticulous control over the disease. Some of the factors which decrease the motivation for self-care are feelings of failure, hopelessness, and disorders of psychosocial health (Polonsky, 2002). Nonetheless, the level of hope and the fostering thereof are effective methods for coping and improving the self-care behaviors (Faller, Bülzebruck, Schilling, Drings, & Lang, 1997). In relation to this, Lloyd et al. (2009) found a significant positive relationship between hope and adherence to a therapeutic regimen and blood glucose control (Lloyd, Cantell, Pacaud, Crawford, & Dewey, 2009). In a spiritual aspect, hope is often accompanied by physical and spiritual health and is related to the concept and value of life; it is considered as an important aspect of human development and enables people to cope with stressful situations and maintain their quality of life (Stephenson, 1991; Benzein, Norberg, & Saveman, 1998; Rustøen, Wiklund, Hanestad, & Moum, 1998). Hope is a dynamic and multi-dimensional force of life which is not determined by uncertain expectations, but is an indefinite expectation of future success (Dufault & Martocchio, 1985). Hope is particularly important among people who are in life-threatening situations or those with unknown outcomes (Gestel-Timmermans, Van Den Bogaard, Brouwers, Herth, & Van Nieuwenhuizen, 2010). Default (1981) studied different dimensions of hope and their influence on the recovery of patients with chronic illnesses, and stated that, “understanding dimensions and spheres of hope help nurses to be hope sources for patients” (Dufault & Martocchio, 1985). He added that, “hope has two spheres: generalized, which encourages continuation with life responsibilities, and particularized, which is related to a special, valuable outcome of life.” He believed that hope is consisted of six dimensions, namely, affective, cognitive, behavioral, affiliative, temporal, and contextual. There are special strategies which are appropriate for improving each of these dimensions. These six dimensions are integrated and made up of three subscales, namely, the cognitive-temporal, affective-behavioral, and affiliative-contextual subscales, which constitute the frameworks for producing elements of the Herth Hope Scale (Dufault & Martocchio, 1985). Obayy et al. (1982) described hope as a pentagon, comprised of consistency in religion, resistant belief, a positive self-concept or high self-esteem, social support, lack of financial problems, and the anticipation of a good and successful future (Obayuwana & Carter, 1982). Meanwhile hopelessness has long been considered a nursing diagnosis in North American nursing diagnosis association. For Nowotny (1989), hope is a measurable quality which could be used in the assessment stage of the nursing process and in the subsequent intervention that seeks to strengthen individuals’ hope and foster it (Nowotny, 1988). Several literatures have focused on the concept and meaning of hope and factors affecting it (Flemming, 1997; Hendricks-Ferguson, 1997; Pourghaznein, Hoshmand, Talasaz Firouzi, & Esmailli, 2003).

Religious beliefs are one of the factors that affects levels of hope. In a study by Herth (1990), dying patients said that their religious beliefs facilitated meaningful feelings about the disease and a change in their understanding, and fostered hope (Herth, 1990). Studies conducted after the 1980s showed that religious beliefs are, in fact, beneficial to individuals’ mental health (Bahrami Ehsan & Tashak, 2004), and that, seemingly, praying and supplication have an effect similar to that of meditation (Meisenhelder & Chandler, 2000). Religious beliefs such as praying may improve a sense of control over stressful events. Furthermore, they provide a sense of purpose and meaning for events which are apparently meaningless (Tarakeshwar, Vanderwerker, Paulk, Pearce, Kasl, & Prigerson, 2006), thereby strengthening the sense of belonging to the whole and to the universe (Mackenzie, Rajagopal et al., 2000). Studies have found that spiritual beliefs influence health habits and behaviors in a special way (Daaleman, Cobb, & Frey, 2001), after controlling for the effects of social, demographic, and non-religious variables; religious coping strategies affect mental health and lead to better physical health (Koenig, Cohen et al., 1995; Tix & Frazier, 1998). As noted earlier, several studies consider religious beliefs an important coping mechanism for patients with chronic diseases. However, little attention is paid to the type of religious coping—positive or negative—that could influence health differently. Positive religious coping methods include praying, positive appraisal of negative situations, feelings of a safe relationship with God, believing in a purposeful life, and hoping for God’s help. In contrast, negative religious coping includes the attribution of difficult situations to divine punishment and feelings of withdrawal. In positive coping, individuals focus on God and hope for his help when facing stressful events (Tarakeshwar et al., 2006). Studies which differentiate between positive and negative religious have indicated that improved mental health is related to more usage of positive strategies (Pargament et al., 1994). In contrast, mental distress is accompanied by the use of negative strategies (Jenkins, 1995; Thompson & Vardaman, 1997; Exline, Yali, & Lobel, 1999). Tarakeshwar’s (2006) study showed that the use of positive religious coping is accompanied by high quality of life in all dimensions (Tarakeshwar et al., 2006). Despite repeated emphasis on the importance of hope in relation to chronic diseases, most studies have primarily investigated hope and factors affecting it, in relation to cancer. A literature search revealed that no studies have been conducted on diabetic patients in Iran. Therefore, the present study aimed to identify the level of hope and its relationship with religious coping in patients with Type 2 Diabetes.

## 2. Materials and Methods

A total of 150 patients participated in this correlational, cross-sectional study involving Type 2diabetes patients, who had been referred by the Karaj Diabetes Associations. The study was conducted from March to June, in 2011. The sample size was determined to be 110, based on a calculation of the correlation coefficient for hope and religious coping, which was obtained from a pilot study. A confidence level of 95% and an exactness of 0.5 were assumed. Since the relationship between hope and demographic variables was also studied, this study was conducted on 150 patients who had been selected through purposive sampling. The inclusion criteria were as follows: giving informed consent, being Iranian, having completed, at least, the fifth elementary grade, being more than 18 years old, having had a history of Type 2 diabetes for at least one year, having a medical file at the Diabetes Clinic, taking anti-diabetic tablets with or without insulin therapy, not being pregnant, and being mentally alert. The exclusion criteria were cognitive disorders which could prevent the completion of questionnaires.

The data were collected through a three-part questionnaire including demographic data (11 items), the Herth Hope Index, and a short measure of religious coping. The Herth Hope Index had 12 items which could be scored on a 4-point Likert scale (i.e., strongly agree, agree, disagree, and strongly disagree). The index is an abridged form of the Herth Hope Scale, which includes 30 items and six conceptual dimensions of hope, which are presented by default. The six dimensions of hope are integrated and make up three subscales, namely, the cognitive-temporal, affective-behavioral, and the affiliate-contextual subscales, as a framework for the items on the Herth Hope Scale. The Herth Hope Index also consists of three subscales comprising four questions each; the scores range from 12 to 48. Higher scores mean higher hope levels. Permission to use this scale has been granted; the scale was developed in 2001 by Ann Herth, a researcher from Kaye. This tool has been used in different countries, for chronic diseases such as cancer, heart failure, kidney transplantation, and diabetes. After translation, ensuring compatibility between each question and its related dimension, and modifying the scale according to the recommendations of experts at Mashhad Ferdowsi University, Department of Psychology, the instrument’s validity was confirmed, using content validity. The instrument’s test-retest reliability was confirmed and a Pearson’s correlation coefficient of 0.82 was obtained, which confirmed its interval consistency. A Cronbach’s alpha of 0.78 was obtained, thus, confirming its internal consistency. In their study, Abdi et al. (2009) standardized the Herth Hope Index, the Herth Hope Scale, and the Miller Hope Scale in a community speaking the Farsi language; all of the questionnaires had acceptable levels of validity and reliability (Abdi & Lari, 2011). The short version of religious coping consists of two subscales, namely, the positive items of a multi-dimensional measure of religiousness/spirituality (MMRS) and the negative items of a brief religious coping measure (RCOP). Each part consisted of seven questions, with a 5-point Likert scale ranging from 1 “strongly disagree” to 5 “strongly agree.” Its validity was confirmed, using content validity and the reliability of the positive religious coping subscale, based on a Cronbach’s alpha of 0.82, while a Cronbach’s alpha of 0.78 was obtained for the negative religious coping subscale. These two subscales showed a significant negative relationship (r=-0.843, p=0.000).

A permission letter was prepared for data collection. After the goal of the study and questionnaire instructions had been explained to the subjects, they were assured that their information would be kept confidential and informed consent was obtained from them, following which, they were selected on the basis of their adherence to the inclusion criteria.

One researcher collected the data from all of the subjects, to ensure that similar information was given to all subjects. The data were analyzed through descriptive and analytic statistics, including Pearson’s correlation coefficient, for the detection of the relationship between the quantitative variables, a T test and a one-way ANOVA, so as to observe differences between the means of hope and religious coping, according to demographic data, and multiple regression analysis, for determining the direct real effect, using SPSS 11.5 statistical software (SPSS Inc., Chicago, IL).

## 3. Results

Of the 150 patients in this study, most were female (61.1%), housewives (49%), married (73.3%), experiencing complications (59.2%), and with an educational level that is below a high school diploma (58%) and a familial history of diabetes (72.6%). The subjects’ mean age was 55.5 years (SD ±10.42) and the mean duration of a diabetes diagnosis was 11.05 years (SD ±8.06).

A hope score of 34.89±8.75 was obtained and most of the subjects (46.7%) showed a high level of hope.

Moreover, a positive religious coping score of 29.59±5.62 and a negative religious coping score of 13.51±4.73 were obtained. The results showed a strong positive relationship between hope and positive religious coping (r=0.876, p=0.000). An increase in positive religious coping was associated with an increase in hope fostering levels, however, an increase in negative religious coping was associated with a decrease in hope levels (r= - 0.239, p=0.003). There was a significant negative relationship between hope and age (r= - 0.373, p=0.000), such that older people obtained lower hope scores, and a significant positive relationship between hope and social support (r=0.659, p=0.000). There were significant differences in the means and standard deviations of hope, based on some demographic characteristics; a post hoc test showed a significant group difference (Table 1).

**Table 1:** Mean score and standard deviation of hope according to some demographic variables

Variables	Hope Mean ± SD	Result of Test
**Marital**			
Married	38.50±7.34	df: (2,146)
Single	36.11±7.20	f:54.29
Widow	24.16±2.7*	p:0.000
**Education**		
less than a high school diploma	33.22±9.20*	df: (2,147)
Diploma	38.27±7.01	f:7.92
Academic	39.63±7.11	p:0.001
**Diabetes complications**		t:-3.60
Yes	33.31±8.82	df:145
No	38.41±7.87	p:0.000
**Job**		
Housewife	33.66±8.80	
Employee	41.72±5.87*	df: (3,143)
Self-employed	33.85±9.85	f:7.92
Pensioner	35.96±7.68	p:0.001

However, no significant difference was found in the level of hope according to other variables such as age, disease duration, familial history, type of treatment being received, the number of people living with the patient, and financial status. A significant relationship was found between positive religious coping and age (r=-0.420, p=0.000), and between positive religious coping and social support (r=0.599, p=0.000). Significant differences were found in the means obtained for positive religious coping, according to some demographic variables (Table 2).

**Table 2:** Mean scores and standard deviations for positive religious coping according to some demographic variables

Variables	Positive religious coping Mean ± SD	Result of Test
**Marital**		
Married	31.34±4.65	df: (2,146)
Single	28.88±7.45	f:36.59
Widow	23.20±3.33*	p:0.000
**Education**		
less than a high school diploma	28.34±5.74*	df: (2,147)
Diploma	31.95±3.77	f:8.36
Academic	31.68±5.55	p:0.000
**Diabetes complications**		t:-3.01
Yes	28.66±5.67	df:138.105
No	31.30±4.86	p:0.003
**Job**		
Housewife	28.87±5.56	
Employee	33.20±2.08*	df: (3,143)
Self-employed	27.75±7.21	f:5.65
Pensioner	30.86±4.58*	p:0.001

However, no significant differences were found on the basis of sex, married life, disease duration, and financial status.

A significant positive relationship was found between negative religious coping and age (r=0.374, p=0.000), whereas a significant negative relationship was found between negative religious coping and social support (r=-0.532, p=0.000). Significant differences were found in negative religious coping, according to some demographic variables (Table 3); however, no significant differences were found for sex, complications, and job.

**Table 3:** Mean scores and standard deviations for negative religious coping according to some demographic variables

Variables	Negative religious coping Mean ± SD	Result of Test
**Marital**		
Married	12.16±3.92	df: (2,146)
Single	14.55±5.52	f:27,472
Widow	18.33±4.03*	p:0.000
**Education**		
Less than a high school diploma	14.62±5.09*	df: (2,147)
Diploma	12.05±3.51	f:6.049
Academic	11.82±4.28	p:0.003

Positive religious coping, social support, level of education, married life, disease-related complications, and job, significantly influenced the level of hope. In order to detect the real effects of these variables were all entered into a regression model; the qualitative variables were converted to indicator variables. Positive religious coping, married life, and social support had considerably more significant effects on the level of hope (R=0.897, p=0.000). The level of education, disease-related complications, and job were excluded from the Model (Table 4).

**Table 4:** Summary results of the multiple regression analysis on positive religious coping, social support, level of education, diabetes complications, job, and marital variables

Variables	Hope		

	B	T	P
positive religious coping	1.26	18.286	0.000
social support	0.387	4.311	0.000
Marital	- 1.047	- 2.654	0.009
Constant coefficient	- 5.919	- 3.437	0.001

## 4. Discussion

It is important for nurses to understand the concept of hope, as it plays an important role in the ability to cope with chronic disease and is an effective mental intervention (Stephenson, 1991). According to El-Gamel, “Herth hope index provides deep signs of patients’ belief about hope and coping skills. “Being able to detect the level of hope could enable nurses to focus on coping strategies (El-Gamel, 1993). In the present study, a hope score of 34.89±8.75 was obtained. A hope score of 40.46 ± 4.88was obtained in a study by Balsanelli et al. (2011), which was conducted in Brazil on diabetic patients. This could likely be due to the different factors affecting hope, such as age, married life, level of education, financial status, social support, religious beliefs, the number of people living with the patient, teaching, and consultation (Herth, 1990; Hwang, Ku et al. 1996; Mun Hong & Ow, 2007).

In the present study, 53.3% of patients showed low and average levels of hope. This result requires consideration, as it indicates low levels of optimism regarding the future, and the feelings accompanying hopelessness could increase the severity of a disease and cumulatively decrease the level of engagement in self-care interventions (Campbell, 1987). Several studies have shown the important role played by hope in chronic diseases and the significant roles of nurses in maintaining and fostering the level of hope (Herth, 1990; Balsanelli, Grossi, & Herth, 2011). There are nursing strategies for each dimension of hope, which means that nursing strategies have the potential to foster hope. Affective strategies include empathy, understanding of discomfort, the patient’s experience of fear and doubt, supportive patience, and paying attention to the patient and his or her family.

In relation to the cognitive dimension, nurses could be helpful by presenting information to patients, resolving misunderstandings and changing misconceptions, and using other patients as role models. In relation to the behavioral dimension, nurses could create a balance between patients ’dependence and independence, as well as develop their self-concepts and enable them to implement self-care. With regard to the temporal dimension, nurses could attach meanings to previous failure and success, and consider patients’ previous experiences. With regard to the contextual dimension, different life situations that might have had a special effect on hope could be studied. Implementation primarily involves the creation of an atmosphere facilitating the achievement of goals, serving as, for example a reminder of values, as well as meaningful and hurtful events relating to life and death. With regard to the affiliative dimension, a nurse should provide opportunities for patients to feel that they were loved and cared for, and attended to. Patients are important and so is their health to others (Dufault & Martocchio, 1985).

As stated earlier, when integrated, the hope dimensions make up three subscales. The affiliate-contextual subscale determines dependency and communication between oneself and others, the self and the soul, and communication with God as a transcendental power (Dufault & Martocchio, 1985). In the present study, following the exclusion of variables affecting hope, positive religious coping was shown to have a strong positive relationship with the level of hope and was indicated as a precursor to development. Negative religious coping was associated with a significant decrease in the levels of hope. These findings are compatible with those of Tarakeshwar (2006); in his study positive religious coping resulted in considerable improvement in quality of life and the physical symptoms of cancer, while negative religious coping resulted in poor quality of life among these patients (Tarakeshwar et al., 2006). Several studies have shown people with strong religious beliefs obtaining significantly higher scores on hope (Ringdal, 1995; Hwang, Ku et al., 1996, Pourghaznein et al., 2003). For many people, personal communication with God when experiencing problems and difficulties provides a sense of calmness, support, and hope. However, for people who use negative religious coping, such as being angry with God and feeling rejected by Him, such communication brings about mental distress (Exline et al., 1999; Pirutinsky, Rosmarin, Pargament, & Midlarsky, 2011). Studies conducted on Christians have shown that spiritual struggles are accompanied by mental problems (Smith, McCullough, & Poll, 2003; Ano & Vasconcelles, 2005). Other studies conducted on Jewish Orthodox subjects showed that all levels of negative religious coping result in depression and spiritual struggles, which leads to depression (Pirutinsky et al., 2011).

In the present study, a significant negative relationship was found between age and hope (p=-0.373, p=0.000), such that advanced age resulted in a significant decrease in the hope score. The relationship between hope and age is controversial. Herth (1990, 1992) and Pourghaznein (2003) have reported no significant relationship between age and level of hope (Herth, 1990, 1992; Pourghaznein et al., 2003); however, Ringdal (1995) found that advanced age results in various degrees of hopelessness.

The above point indicates a need for more attention to be paid to elderly individuals. According to this study and others, married people and people with high levels of social support show high levels of hope. According to some studies, social isolation strengthens the feeling of hopelessness, and patients displaying strong communication with their relatives showed high levels of physical and psychosocial health (Herth, 1992; Ballard, Green, McCaa, & Logsdon, 1997). In this study, patients who had disease-related complications showed significantly low levels of hope; this finding indicates a need for attention to be paid in this regard.

## 5. Conclusions

As to In the present study, most of the diabetic patients showed low and average levels of hope. In the Muslim, as well as Christian, and Jewish Orthodox communities, positive religious coping has a significant effect on the level of hope, whereas negative religious coping results in spiritual struggles and mental distress. Moreover, married life and social support lead to a significant increase in the level of hope. However, a significant positive relationship was found between positive religious coping and social support, therefore, strong social support could result in high positive religious coping and the fostering of hope. These would enable effective coping with the disease, an increase in self-care, adherence to the therapeutic regimen, a decrease in complications, and improved quality of life. It is recommended that more studies be conducted on factors affecting positive religious coping among individuals with chronic diseases.

The level of hope in patients has been studied and the authors propose studding the level of hope in nurses and its relationship with care.

**Limitations of Study**

The samples of this study are selected non-randomly and ethically only those who tended to participate were selected. It’s obvious that level of hope of our samples is different from those who didn’t participate.
